# What Is “Socioeconomic Position (SEP),” and How Might It Modify Air Pollution-Health Associations? Cohering Findings, Identifying Challenges, and Disentangling Effects of SEP and Race in US City Settings

**DOI:** 10.1007/s40572-022-00359-3

**Published:** 2022-05-05

**Authors:** Jane E. Clougherty, Jamie L. Humphrey, Ellen J. Kinnee, Richard Remigio, Perry E. Sheffield

**Affiliations:** 1 Department of Environmental and Occupational Health, Drexel University Dornsife School of Public Health, Philadelphia, PA USA; 2 Center for Health Analytics, Media & Policy, RTI International, Research Triangle Park, NC 27709 USA; 3 University Center for Social and Urban Research (UCSUR), University of Pittsburgh, Pittsburgh, PA USA; 4 Maryland Institute for Applied Environmental Health, School of Public Health, University of Maryland, College Park, MD USA; 5 Environmental Medicine and Public Health, and Pediatrics, Icahn School of Medicine at Mount Sinai, New York, NY USA

## Abstract

**Purpose of Review:**

Environmental epidemiology has long considered socioeconomic position (SEP) to be an important confounder of pollution effects on health, given that, in the USA, lower-income and minority communities are often disproportionately exposed to pollution. In recent decades, a growing literature has revealed that lower-SEP communities may also be more susceptible to pollution. Given the vast number of material and psychosocial stressors that vary by SEP, however, it is unclear which specific aspects of SEP may underlie this susceptibility. As environmental epidemiology engages more rigorously with issues of differential susceptibility, it is pertinent to define SEP more clearly, to disentangle its many aspects, and to move towards identifying causal components. Myriad stressors and exposures vary with SEP, with effects accumulating and interacting over the lifecourse. *Here, we ask: In the context of environmental epidemiology, how do we meaningfully characterize”SEP”?*

**Recent Findings:**

In answering this question, it is critical to acknowledge that SEP, stressors, and pollution are differentially distributed by race in US cities. These distributions have been shaped by neighborhood sorting and race-based residential segregation rooted in historical policies and processes (e.g., redlining), which have served to concentrate wealth and opportunities for education and employment in predominantly-white communities. As a result, it is now profoundly challenging to separate SEP from race in the urban US setting.

**Summary:**

Here, we cohere evidence from our recent and on-going studies aimed at disentangling synergistic health effects among SEP-related stressors and pollutants. We consider an array of SEP-linked social stressors, and discuss persistent challenges in this epidemiology, many of which are related to spatial confounding among multiple pollutants and stressors. Combining quantitative results with insights from qualitative data on neighborhood perceptions and stress (including violence and police-community relations), we offer a lens towards unpacking the complex interplay among SEP, community stressors, race, and pollution in US cities.

## Introduction

Environmental epidemiology has long considered socioeconomic position (SEP) to be an important confounder of pollution impacts on health. This concern is well-founded, as pollutants are often higher near major sources (e.g., industry, vehicular traffic), which are disproportionately located in lower-SEP communities [1]. The environmental justice movement emerged as response to the inequitable environmental burdens faced by those communities [2]. Over the past two decades, a growing literature has also revealed a greater susceptibility to pollution in lower-SEP communities [3]. Given the complexity of SEP as a construct, however, and the vast number of material and psychosocial stressors which vary along the SEP gradient, it remains unclear which specific SEP-related stressors may underlie this apparent susceptibility, and how such interactions may differ for various pollutants, stressors, or health outcomes, across varied populations.

Environmental epidemiology is engaging more rigorously with questions of differential exposure and susceptibility by SEP. As a result, it becomes pertinent to define this construct more clearly, and to consider which specific aspects of SEP may be responsible for observed confounding and effect modification. Traditionally, SEP has been measured using well-established indicators (e.g., education, wealth, income, job grade), but the true range of stressors that vary by SEP is immense (e.g., housing insecurity, job strain, food insecurity, violence, etc.). They invariably accumulate and interact over the life course. *Here, we ask: In the context of environmental epidemiology, how do we meaningfully characterize”SEP”?*

In this paper, we aim toward a richer understanding of SEP, for the purposes of environmental epidemiology, by cohering evidence from qualitative and quantitative studies, largely focused in New York City (NYC), as an exemplar of US urban settings. *Importantly, we hope to demonstrate, that—as is the case in most US cities—race (a social construct with overt and insidious policies and practices) and SEP are so intimately intertwined that their etiological effects are often difficult to distinguish. Moreover, racial disparities in exposures to SEP-related stressors—often stemming from race-based residential segregation* [4–7]*—may profoundly shape disparities in pollution susceptibility.*

Weaving together results from multiple qualitative and quantitative studies, we hope to provide novel insights on the complex relationships and interactions among SEP, social stressors, race-based residential segregation, and air pollution, as related to health. First, we present an overview of the construct of SEP. Second, we discuss how SEP manifests in chronic social stressor exposures, which subsequently impact health. Third, we address the complex relationships among SEP and race—which are intertwined in American society—and how racial segregation perpetuates racial stratification in SEP, pollution exposures, and, ultimately, health disparities. Fourth, we present several pressing theoretical and methodologic challenges ahead in the study of SEP effects in environmental epidemiology. Finally, we conclude with a call for more solutions-oriented research with an emphasis on assets, resilience, and opportunities for improving health—by addressing specific stressors—in lower-SEP communities, and for reducing health disparities.

## First, What Is Socioeconomic Position (SEP)?

In Sociology, Socioeconomic Status (SES) or SEP “refers to the social and economic factors that influence what positions individuals or groups hold within the structure of a society” [8] and “reflects one’s access to collectively desired resources.” [9] In Social Epidemiology and Health Geography, SEP is understood to describe not only tangible access to goods and services, but also the prestige and sense of identity this privileged access represents. In essence, SEP is a complex construct entailing aspects of relative material and psychosocial (dis)advantage—accumulating and interacting over the lifecourse—and the societal structures and norms that underpin and perpetuate deprivations and inequities (e.g., racism, sexism).

In air pollution epidemiology, we have traditionally “adjusted for” SEP, in hopes of accounting for its myriad exposures and life conditions (e.g., health care access, income, smoking, diet, education, occupational exposures, job insecurity, housing insecurity, indoor exposures, violence, noise, police-community relations, direct experiences of racism or sexism). These stressors are often confounded among themselves; however, complicating our understanding of how each component may affect sensitivity to air pollution or other physical agents. Further, these stressors can aggregate and interact over the lifecourse, and even across generations through socioeconomic (e.g., wealth generation) and biologic (e.g., DNA methylation) pathways [10–13].

All of these dimensions of SEP are products of larger societal forces, and serve as both physical manifestations of predominant power structures, and continual reminders of one’s social position. That is, a refinery located in a low-income community both directly exposes the population to volatile organic compounds (VOCs), and also, as an eyesore and source of odors, continually reminds community members that their health and well-being may not be valued by the company, by city decision-makers, or by the larger society [14–16].

It is important to note that, unlike air pollution itself (which is physical and tangible), SEP is inherently *relational;* it reflects an individual’s *relative* standing within a given society, and may change, to varying extents, over the life course, through actions of individual agency (e.g., gaining education), or societal shifts (e.g., evolving gender norms, equality for LGBTQ communities).

It is beyond the scope of this paper to review the many commonly-available SEP indicators (e.g., income, education, wealth), and their relative strengths and weaknesses (See Hajat et al., EHP, 2021: Table 1) [17]. We do, however, wish to encourage environmental epidemiologists to carefully consider, when selecting SEP indicator(s): (1) The hypothesized mechanisms to health or susceptibility; (2) Limitations and biases inherent to each indicator (e.g., educational norms have varied over time, and thus variation in the metric varies by cohort age and racial discrimination in data collection can further bias data) [18]; and (3) Scale and resolution, especially as compared to resolution in environmental exposure metrics.

## How Does SEP Impact Health and Susceptibility?

The Social Epidemiology literature has demonstrated a consistent gradient in health across the SEP spectrum, with higher-SEP individuals and communities enjoying, on average, better health and longevity. Importantly, this “social gradient in health” replicated across a wide range of health outcomes [19–21], suggests that the mechanisms through which SEP operates are broad in scope, impacting multiple bodily systems, and its impacts are not restricted to those at the lowest end of the gradient through tangible material or resource deprivation (e.g., food insecurity, lack of access to health care). The persistence of a social gradient in health across societies, and even in non-human primates [22, 23], has prompted serious attention to the health impact of social status *in and of itself*, rather than the material resources it confers, giving rise to psychosocial (vs. material) explanations for this gradient.

Psychological stress—defined as the *perception* that challenges in one’s life are overwhelming to one’s abilities and resources to meet those challenges [24]—has emerged as a leading explanation for this persistent social gradient in health [25]. While acute stress responses evolved to provide short-term physiologic benefits facilitating energy availability (e.g., epinephrine production, bronchodilation), chronic stress (stress that is that is recurrent or prolonged) dysregulates normal acute stress function, and leads to physiologic wear-and-tear. Chronic stress has been associated with outcomes as varied as neuroendocrine dysfunction [26–28], impaired wound healing [29], susceptibility to the common cold [30], slowed growth rates in children [31], telomere shortening [32, 33], and susceptibility to ionizing radiation [34].

## Community Stressors as SEP Indicators

There are substantial challenges in measuring stress for epidemiologic purposes, however. Because stress is a construct based on *perception,* a challenge that is perceived as stressful to one individual may not be stressful to another. Stress is ideally measured using self-reports, capturing individual appraisal. For large cohorts and administrative databases (e.g., electronic medical records), however, individual interviews or surveys are often infeasible. As a result, large epidemiologic studies must often rely on community stressors (e.g., violent crime) or other indicators of stressor exposures, as proxies for perceived stress. It is important, in such cases, that indicators be validated against individual appraisals or perceived stress measures, and within- vs. between-community variance be carefully considered.

In New York City, we attempted to understand the relationship between community stressors and individual-level stress. Specifically, we examined an array of community stressors [35] and performed citywide focus groups to identify stressors prioritized by diverse communities [36]. We then developed composite socioeconomic deprivation indexes leveraging the spatial patterns across these multiple stressors [37, 38]. Finally, we implemented citywide surveys to assess individual-level constructs related to perception (i.e., perceived exposures to community stressors, perceived neighborhood disorder, perceived stress, perceived police-community relations, and mental health), with a geographic information systems (GIS)-based mapping interface allowing survey respondents to delineate the unique area that they consider their “neighborhood” [39].

Violent crime stood out as a paramount stressor of concern across all NYC neighborhoods—it was the only community stressor emphasized by every focus group [36], it has most consistently explained variation in perceived stress [40], and more strongly modified relationships between air pollution and health than did material deprivation [41, 42]. Importantly, violent crime may *not* be the most important stressor in every city or setting—rather, *we emphasize that investigators need to take the time to identify and understand the stressors and stress indicators most salient in their specific setting and population of interest.*

## The Inextricable Relationships Among SEP, Race, and Susceptibility in US Cities

Race and ethnicity—which are social, rather than biological, constructs—are intricately entangled with SEP in U.S. cities. Almost all aspects of SEP are differentially distributed by race and ethnicity, including those stressors that we discuss here as primary drivers of SEP-related susceptibility to pollution (e.g., violence, poverty). While disparities exist for many historically marginalized racial and ethnic groups in the U.S., we focus here on the contrast of Black and White populations, but still acknowledge the importance of unpacking the SEP/race relationship for other groups and subgroups, as disproportionate health burdens for other subgroups remains an important gap in the literature [43]. These racial disparities in stressor exposures stem from both historical practices (e.g., redlining) and modern processes of segregation, which concentrate wealth in White communities, and limit economic advancement for families of color. Today, the average U.S. Black family has less than 15% of the average White family’s wealth [44]. Racial disparities in spatially-distributed exposures—including pollution and community stressors—however, are not entirely explained by wealth [45–47], as evidenced by research showing that Black Americans are less likely to move to a new neighborhood following a personal financial gain (e.g., promotion or raise), than are Whites, and experience lesser residential mobility, on average, over the lifecourse [48, 49]. Such findings speak to ongoing exposures to social stressors (e.g., direct experiences of discrimination), and/or a low sense of safety for Black populations (stemming from anti-Black policing, or biased real estate practices), that reinforce racial segregation despite greater economic equity, and re-affirm the role of persistent neighborhood exposures in shaping racial health disparities.

**Fig. 1:**
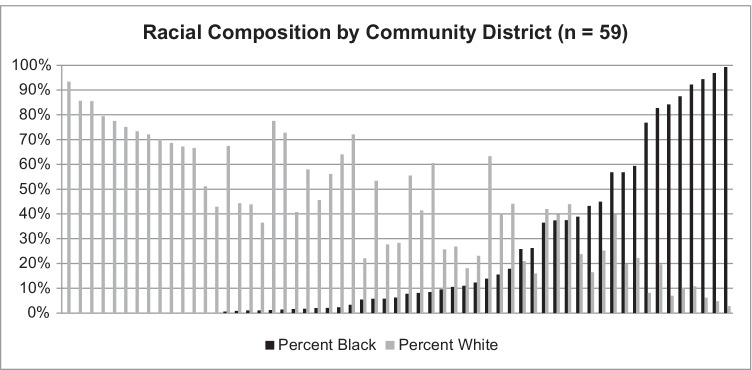
Most NYC Community Districts (*n* = 59) are either predominantly-White (grey bars) or predominantly-Black (black bars). Even in this relatively diverse city, very few neighborhoods are evenly mixed

**Fig. 2:**
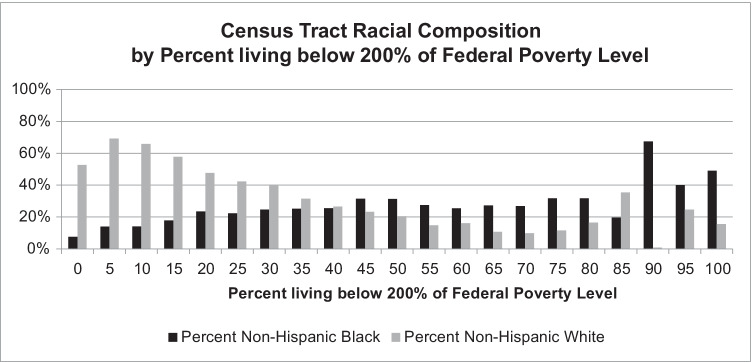
Predominantly-white census tracts are substantially wealthier (have a smaller percentage of poor residents) than are predominantly-Black census tracts in NYC (*n* = 2,167)

Even in NYC, among the most diverse U.S. cities, segregation is deeply entrenched, and most census tracts/districts are predominantly-White or -Black; very few are truly mixed (Fig. 1). Critically, this lack of crossover complicates the study of segregation and neighborhood effects on health, by statistically hampering our ability to differentiate effects of neighborhood-level SEP from individual-level race. As shown in Fig. 2, a larger percentage of those living in wealthy (low-poverty) census tracts are White, and a higher percentage of the population in high-poverty tracts is Black. In our data including all cardiovascular disease (CVD) admissions at NYC hospitals for 2005–2011, only 10% of cases from the most-affluent quintile of tracts are Black; only 18% from the least-affluent quintile are White. Any cross-level analysis is statistically under-powered (too few cases living in the “opposite” neighborhood) and may be inherently non-representative, if Blacks living in predominantly-White neighborhoods are relatively affluent, and Whites living in Black neighborhoods are relatively poor, compared to others of their racial group. This lack of overlap also precludes propensity stratification or similar analyses, given too few truly 'mixed’ communities, or individuals who might plausibly live in either neighborhood type.

### Stress and Race

We noted in the prior sections that SEP likely influences health via stress mechanisms, as well as through physical or material pathways. We further showed that violence is a paramount stressor in NYC, and among the strongest community-level predictors of perceived stress. We need also acknowledge that, in NYC and most other U.S. cities, there are stark racial disparities in neighborhood violent crime rates, with Black Americans consistently experiencing higher exposures. In recent decades, though violent crime has declined overall, predominantly-Black neighborhoods (those > 70% Black) account for 94% of those with increasing homicide rates [50]. In our citywide survey data, objective and perceived neighborhood violence was significantly higher for Black than White respondents. Specifically, across 2,167 NYC census tracts, violent crime—as defined by police report data—was 150% higher in predominantly-Black [mean = 66.7 events/ 10,000 persons annually (SD = 41.4)] vs. predominantly-White tracts [mean = 26.6 (SD = 57.2)]. In the hospitals CVD data, 70% of patients from the safest quintile of tracts were White, vs. only 10% from the least-safe quintile. Recent studies have noted that over-policing in Black communities may drive up official crime rates [51]; thus, we consider both ‘objective’ and ‘perceived’ crime, and restricted this analysis to the most thoroughly-reported crimes (i.e., murder, felony assault).

Also from our city-wide survey data, neighborhood violent crime and personal victimization were separately associated with higher perceived stress, anxiety, and depression, after adjusting for participant age, sex, race, ethnicity, neighborhood tenure, survey format, and season. [i.e., A 1-IQR increase in neighborhood violent crime conferred higher odds of reporting above-median perceived stress [adj OR = 1.14 (95% CI = 0.97–1.33)], as did reporting a personal experience of victimization in the neighborhood [adj OR = 1.92 (1.45–2.52)]. Importantly, both associations were significantly attenuated by having a positive perception of local community-police relations (*p* = 0.007). But, because Black Americans experience more violence at the hands of police, and may have less trust in police overall (in our survey, only 20% of Blacks reported a positive perception of local police-community relations, vs. 60% of Whites), the communities most impacted by violence may be least likely to benefit from a greater law enforcement presence.

Relationships among race, racism, and perceived stress should not be over-simplified. Some evidence suggests *lesser* response to acute stressors, but greater long-term depression and chronic stress, in marginalized racial groups [52, 53]. For example, a recent survey of unpaid caregivers to elderly or ill persons (76% female) found that those in environmental justice areas reported *lower* levels of unmet needs, depression, and poor mental health, possibly pointing to resilience and coping skills developed over time, [54] or personal social support. In our survey, despite greater neighborhood violence exposures among Blacks, violence did *not* more strongly predict perceived stress for Blacks than for Whites, nor for women vs. men, contrary to our hypotheses. Notably, however, the opposite has been observed in children; studies of impacts of neighborhood violence on children’s cognition and mental health report stronger impacts in children of the same race as the victim, who are disproportionately Black [55] [in NYPD data, 67.8% of homicide victims from 2006–2019 were Black [56]].

### Race, Pollution, and Susceptibility

These issues of SEP, stress, and race may be exemplified in the study of cardiovascular disease (CVD), the leading cause of death in the USA [57]. CVD disproportionately impacts Blacks, who develop CVD at younger ages, on average. In NYC, the median age at CVD event was 10 years younger for Blacks than Whites (65 vs. 75 years old); for heart failure, the median age for Blacks was 14 years younger (67 vs. 81 years old). In race-stratified case-crossover analysis of air pollution impacts on CVD event risk, we found similar responses to daily pollution increases in both races—though these responses were occurring at much younger ages for Blacks than for Whites. In essence, *while air pollution predicted which day someone had a heart attack, race predicted which decade* [37, 42].

This racial discrepancy in years of healthy life emphasizes the need for analyses capturing the longer time scales relevant to social processes underlying susceptibility. Persistent discrimination and accumulated life stressors operate over years and decades—the analysis of which is amenable to lifecourse approaches, rather than the day-to-day variation emphasized in studies of acute environmental exposures (i.e., pollution events) on CVD events and other ‘acute’ health outcomes.

## Challenges in Measuring and Analyzing SEP in Environmental Epidemiology

### Spatial Resolution in Stressors vs Pollution

In epidemiology, SEP and social stressors are often measured with much less spatial and temporal resolution than is pollution. We regularly employ sophisticated fine-scale spatiotemporal models for pollution exposures—combining data from field sampling campaigns, regulatory monitoring, dispersion modeling, satellite imagery, and time-activity data—to create individualized, refined exposure estimates, reducing misclassification in the primary exposure of interest. In contrast, we often adjust for SEP using readily-available census data or other aggregate-level administrative data—generally reported as annual or multi-year averages, often as coarse categorical variables—and expend relatively little effort in exploring the implications of this differential misclassification. To address this discrepancy, we compared associations with CVD rates for a range of social stressors and pollutants *at the same spatial and temporal scale, which requires aggregating the more-resolved metric (time–space pollution) to the less-resolved scale (census tract annual averages)*. After doing so, we found that, in models testing a 1-IQR difference in risk, for every combination of stressor and pollutant against tract-level CVD event rates, mutually-adjusted *associations for pollutants were consistently non-significant, while most stressors retained significance, with much larger magnitudes of association* [37]. Thus, offered comparable spatial and temporal resolution, the social variables explained much more variability in disease risk—raising important questions about the extent to which pollution-CVD associations observed to date have been contingent on tightly-controlled misclassification in the main exposure of interest, with relatively coarse measurement of important confounders.

There is a well-established concept of the “decay curve” in exposure science, which captures the expected decrease in pollutant concentrations with distance from a given source (e.g., roadway) [58, 59]. This concept has supported a vast environmental epidemiology literature by enabling individual-level exposure estimates for large cohorts based on distance to source, quantifiable in Geographic Information Systems (GIS), and undergirding spatial modeling approaches for pollution exposures (e.g., [land use regression (LUR)]. In contrast, space–time methods for social stressors are far less developed, and critical distances in the relationship between, for example, violence and perceived stress are entirely unknown: *Is a violent crime on one’s block or four blocks away equally stressful? For how long following a violent crime are stress and CVD risks elevated in the community? For whom? Are impacts of each violent event weaker/ stronger in communities with higher crime rates? Which individuals or communities are more/ less susceptible to the stress and health impacts of neighborhood crime?* By relying on coarse administrative data for SEP and stressor exposures, we fail to appreciate these meaningful nuances in exposure patterns, and introduce unknown spatial errors. Likewise, crime and other acute stressors vary over time (e.g., violent crime is higher at night, on weekends, and during summer) [60], and the impact of each event may differ by other neighborhood characteristics. Studies of both stressors and pollution are thereby hampered by temporal and spatial misclassification in social metrics, with little research to date detailing the space–time relationships for neighborhood stressors.

### Context vs. Composition

In interpreting community-level SEP and stressor metrics, social epidemiologists and health geographers emphasize the distinction between *context* (a characteristic or essence of the place itself) and *composition* (characteristics of the individuals who inhabit that space). This distinction is increasingly relevant in environmental epidemiology, as we begin to consider interactions among individual stressors (e.g., job strain, victimization), societal stressors operating at the aggregate level (e.g., structural racism), and pollution exposures clustered (autocorrelated) across individuals in the same community, but normally measured, as possible, at the individual level.

These distinctions in level of operation are key to identifying appropriate scales of measurement, and interpretation of mechanisms of action for community stressor impacts on health. For example, in our citywide surveys, we found that a 1-IQR increase in community assault rates conferred twice the odds of a resident reporting feeling unsafe, and twice the odds of perceiving their neighborhood to be high in crime, but no elevated odds of having had a personal experience of violence. The result suggests, importantly, that neighborhood violent crime rates appear to capture something about the “experience” of living in an environment that feels unsafe (a chronic “ambient” exposure), rather than proxying for individual’s own acute crime experience (*unpublished data*).

### Thresholds, Saturation, and Non-linearity in Effect Modification

In a prior review, one co-author reported that most evidence, to that date, indicated greater susceptibility to environmental contaminants with greater stressor exposures or higher perceived stress (i.e., susceptibility in the hypothesized direction) [3]. However, as pollution and chronic stress are separately linked to many health outcomes, individuals with especially high exposure to either stress or pollution may be more likely to experience ill health, regardless of the other exposure. That is, very high exposures to either pollution or chronic stressors may plausibly overwhelm any potential interaction.

Such “saturation effects” require careful attention to the range and distribution of each exposure independently, in the population of interest. Comparing the range of observed exposures to that of the general population may inform on whether interaction or saturation effects should be expected, and how they may be interpreted. For example, in very early work on this hypothesis, one co-author found *less* asthma symptom improvement, in response to allergen-reducing indoor environmental interventions, among children in public housing whose caregivers reported *greater* fear of neighborhood violence [61], suggesting that fear of violence may have outweighed effects of allergens in this highly-susceptible cohort.

Similarly, in recent analysis of modification by categorical tract-level chronic stressors on non-linear relationships between NO_2_ and birthweight in NYC, we consistently found the *lowest average* birthweights in the least-affluent tracts (*lowest-SEP* or *highest-violence)*, as expected. However, apparent negative effects of NO_2_ on birthweight were strongest in the most-affluent tracts (*highest-SEP* or *lowest-violence*). In essence, very high stressor exposures conferred very low average birthweights, with minimal additional impact of NO_2_). In more affluent communities, however, we saw clear negative pollution-birthweight associations, as hypothesized, unencumbered, or not “washed-out,” by other exposures [62, 63]. Finally, we have found that year-round exposures to multiple pollutants may, in some cases, have lesser impact on child asthma exacerbations in very-high violence communities, compared to those in the lowest quintile [64, 65].

### Modification by Multiple Stressors

Stressors are neither randomly, nor independently, distributed. Most lower-SEP communities experience multiple chronic stressors simultaneously, and, even in settings with a strong paramount stressor (e.g., violence), it does not negate the importance of others (e.g., poverty, housing insecurity, food insecurity, structural racism, sexism). As stressors are not independently distributed, and most lower-SEP communities suffer multiple stressors simultaneously [37], testing modification by any one alone almost certainly captures some impact of other correlated stressors (i.e., testing for modification only by crowded housing conditions, for example, almost certainly captures some aspects of modification by food insecurity, to the extent that they are correlated). As such, any observed modification by a single stressor may, in part, stand proxy for modification by other clustered stressors, especially where there is substantial exposure misclassification, as is normally the case where using community-level indicators.

To date, there has been relatively little methodologic attention to the development of multiple-modifier methods. The few environmental epidemiology studies that have examined multiple modifiers simultaneously have done so using separate interaction terms in the same model [66]—though it remains unclear what errors may be induced by repeating the same pollutant term in multiple interactions in the same model (e.g., NO_2_ x violence, NO_2_ x poverty, etc.). This is particularly the case where potential modifiers are spatially confounded, and misclassification in any one may impact observed modification by another. In our study of ozone-asthma associations during summer, we compared modification by violence and a material socioeconomic deprivation index (SDI) using four different approaches—separate interaction models with categorical stress modifiers, separate interaction terms with categorical stressors in the same model, separate continuous interactions in the same model, and median-dichotomized cross-stratified categorical interaction terms (i.e., high-violence/low-poverty, low-violence/ high-poverty, etc.). In each case, modification by violence was consistently stronger and more significant than by poverty, [67] suggesting that violence may be the stronger, or more consistent, modifier, in the NYC setting.

In contrast, in the case of CVD, multiple-modifier results were more complicated. After adjusting for modification by community racial and ethnic composition (due to concerns about segregation and clustered social stressors), we found that, in separate models, NO_2_-CVD associations were significant *only in the highest quintile of violence or poverty*. Testing both interactions, for categorical violence and poverty in the same model, however, increased observed pollution-CVD associations in all quintiles. Further, violence but not poverty, displayed modification in the hypothesized direction, though the trend in NO_2_ effects across violence quintiles was not significant [68, 69].

### Mediated-Modifier Models: to What Extent Is Modification by SEP Attributable to Chronic Stress?

To develop actionable interventions to alleviate SEP-related susceptibilities, there is a need to clearly identify those key social stressors that are the “causal components” of SEP, and to quantify that portion of observed susceptibility by SEP which may be attributable to specific actionable stressors (i.e., mediated-modifier models) [70]. Likewise, Structural Equation Models (SEMs) may be a useful approach to detailing how (i.e., via which stressors) SEP may most strongly impact health and susceptibility, as SEMs can support testing of multiple modifiers in non-linear and continuous forms, in interaction with multiple pollutants—plausibly informing on complex mechanisms and pathways.

No studies, to our knowledge, have specifically aimed to quantify that portion of observed modification by SEP that is explained by (mediated via) perceived stress or specific social stressors. The development and application of such ‘mediated-modifier’ models remains an important methodologic path forward in this epidemiology. Though none of the studies we described here was explicitly designed to resolve this issue, each does inform on it, with differing results.

- In the case of birthweight, we observed significant modification of associations for NO_2_ by both a composite measure of SEP and violence, in separate models. These significant modification effects were somewhat dissipated in models including both modifiers, however, suggesting that modification by SEP was partially, but not entirely, explained by violence [62, 63].
- In the case of summertime ozone and asthma, observed modifications by SEP became non-significant when adjusting for modification by violence, which proved a more consistent, significant modifier—suggesting that much of the modification originally attributed to SEP was due to violence [67].
- Finally, in the case of CVD, in single-modifier models, we observed significant associations for NO_2_ only in the highest-violence or lowest-SEP quintiles. In models including both interactions, however, positive NO_2_-CVD associations increased in all quintiles, suggesting some dampening in observable modification by each stressor until both modifiers were accounted for [68, 69].

None of these studies was designed as a definitive test of the extent to which modification by SEP is explained by violence (though violence presents consistently strong effects, in the NYC setting). Rather, the variation represented by these results underscores the need for stronger methods to test mediation via multiple stressors in the SEP-susceptibility relationship.

## **Future Directions**

### Neighborhoods as Positive Entities Promoting Health and Resilience

To date, more research has explored negative aspects of lower-SEP communities (stressors) in relation to pollution susceptibility. Fewer studies have explored the many neighborhood assets and resources (e.g., daycare centers, supermarkets, recreation centers, pharmacies)—often more prevalent, or of higher quality in higher-SEP communities—which may ameliorate stress- or pollution-related health impacts, with the notable exception of the growing literature on urban greenspace, walkability, and health. Quantifying the benefits of community assets, we are finding, may be more complicated than quantifying impacts of stressors, for many reasons: (1) Mere presence of an asset in a community does not imply access (e.g., a high-end grocery store in a low-income community may not improve local diets, and may exacerbate perceived inequities); (2) Asset quality can determine usability, but is not normally indicated in available data (e.g., unclean parks, broken playground equipment); (3) The multiple physical and psychosocial mechanisms through which assets operate are varied and often difficult to discern (e.g., *To improve health, is it necessary to actually use a local hospital or pharmacy, or is it important to simply know that it is there, available if needed?*); (4) Assets not responsive to community needs are unlikely to be beneficial (e.g., A community with four groceries but no hospital needs not a fifth grocery.); and (5) Depending on audience and context, some assets may act as stressors. To this latter point, sociology and feminist geography research has long documented that many urban women feel unsafe walking in or near parks and greenspaces, especially after dark—curtailing activities, limiting physical activity, and increasing perceived stress [71, 72]. We recently re-analyzed results of a randomized vacant lot greening intervention in Philadelphia and found that, despite decreases in objective violent crime after lot greening, women living nearby felt significantly *less* safe at night, compared to men, post-intervention [73].

## Discussion

The qualitative and quantitative studies, compiled here, and considered as a whole, offer a unique lens towards understanding the complex interplay among SEP, SEP-associated social stressors, race/ethnicity, and pollution exposures in US urban settings. By highlighting examples from one city with excellent data quality and availability, we were able to cohere more streams of information than is typically possible, and examine spatiotemporal relationships at very fine scales. These strengths have enabled us to dive deeply into some nuances of these interactions and their interpretations—in some cases, identifying the limits of our current methods.

We have found, for example, that while air pollution reveals daily associations with CVD event risk, social processes underlying susceptibility operate across years and decades, and thus the effects of each exposure type are optimally captured using very different analytic methods. We have also found that current epidemiologic tools have not helped us to fully capture processes of race-based residential segregation, neighborhood sorting, or disproportionate racial composition across neighborhoods strongly varying in stressor exposures (esp. violence), leading to confounding between individual- and community-level variables, and off-support inference when comparing between racial groups, given their very different distributions in some social stressors. In particular, in NYC, we found that concern about violence was ubiquitous across citywide focus groups, and violent crime was consistently the strongest predictor of inter-community variance in perceived stress—but the distribution in violent crime itself was highly skewed, much higher in predominantly-Black neighborhoods, and with a number of very high outlier neighborhoods, presenting startling disproportionate risk to Black New Yorkers.

In summary, and moving forward, we suggest that researchers pay greater attention to the hypothesized mechanisms linking SEP to health and susceptibility, and use those mechanisms to guide selection and validation of SEP and stressor indicators. We also suggest that more effort towards disentangling processes related to segregation and neighborhood sorting by race/ethnicity, and consider multiple-modifier and mediated-modifier methods to better quantify interactions with multiple correlated stressors, and to test potential interventions. Studies aiming to understand SEP-related susceptibility may benefit from some attention to assets and resilience; some neighborhood amenities can be health-promoting and offset impacts of poor air quality, though, in varying contexts, the same resource may be perceived as either an asset or a stressor (e.g., police presence). Likewise, while race-based residential segregation has led to vast inequities in resource access and concentrated stressors, there may be some key advantages for residents of ethnically-clustered communities, in terms of culture, language, and shared values. Finally, identifying assets and disseminating knowledge via publicly-available tools [e.g., such as EPA’s EJSCREEN [74], an environmental justice mapping tool designed for use by community groups, or asset maps such as those provided by NYCityMap [75] or Western Pennsylvania Regional Data Center [76] may help to translate results into actionable policy, towards improving community health and reducing health disparities.

## Disclaimer

This publication was produced using raw data purchased from or provided by the New York State Department of Health (NYSDOH). However, the conclusions derived, and views expressed herein are those of the author(s) and do not reflect the conclusions or views of NYSDOH. NYSDOH, its employees, officers, and agents make no representation, warranty or guarantee as to the accuracy, completeness, currency, or suitability of the information provided here.
